# Synthetic opioids: a review and clinical update

**DOI:** 10.1177/20451253221139616

**Published:** 2022-12-10

**Authors:** Abu Shafi, Alex J. Berry, Harry Sumnall, David M. Wood, Derek K. Tracy

**Affiliations:** South West London and Saint George’s Mental Health NHS Trust, London, UK; Division of Psychiatry, University College London, London, UK; Liverpool John Moores University, Liverpool, UK; Clinical Toxicology, Guy’s and St Thomas’ NHS Foundation Trust, London, UK; Clinical Toxicology, Faculty of Life Sciences and Medicine, King’s College London, London, UK; West London NHS Trust, Trust Headquarters, 1 Armstrong Way, Southall UB2 4SD, UK

**Keywords:** fentanils, fentanyl, laboratory testing, new psychoactive substances, NPS, opioid crisis, overdose, public health, synthetic opioids

## Abstract

The term ‘opioids’ refers to both the natural compounds (‘opiates’) which are extracted from the opium poppy plant (*Papaver somniferum*) and their semi-synthetic and synthetic derivatives. They all possess relatively similar biochemical profiles and interact with the opioid receptors within the human body to produce a wide range of physiological effects. They have historically been used for medicinal purposes, their analgesic and sedative effects, and in the management of chronic and severe pain. They have also been used for non-medicinal and recreational purposes to produce feelings of relaxation, euphoria and well-being. Over the last decade, the emergence of an illegal market in new synthetic opioids has become a major global public health issue, associated with a substantial increase in unintentional overdoses and drug-related deaths. Synthetic opioids include fentanyl, its analogues and emerging non-fentanyl opioids. Their popularity relates to changes in criminal markets, pricing, potency, availability compared to classic opioids, ease of transport and use, rapid effect and lack of detection by conventional testing technologies. This article expands on our previous review on new psychoactive substances. We now provide a more in-depth review on synthetic opioids and explore the current challenges faced by people who use drugs, healthcare professionals, and global public health systems.

## Introduction

New synthetic opioids are a major public health concern. This narrative review expands on the authors’ 2020 publication on new psychoactive substances (NPS),^1^ and we now provide a more in-depth review on synthetic opioids, including their historical emergence, mechanism of action, mode of use, acute harms, chemical structures, management of acute toxicity and overdoses, dependence and withdrawal syndromes and current challenges faced in laboratory testing. Inevitably, in a single paper such as this, there are limitations to the amount of information that can be provided about individual compounds and their global impact. However, the included relevant representative literature referenced in this paper can provide further reading on the topic.

## Historical emergence

Historical records regarding the analgesic use of the opium poppy plant (*Papaver somniferum*) date back to 3400 BC in Mesopotamia, when the ancient Sumerians extracted opium from the milky sap of the plant and referred to the bright red flowers as ‘the joy plant’.^2–4^ However, it was not until the nineteenth century that the plant’s natural alkaloids and active ingredients started to be systematically isolated and analysed, leading to the discovery of morphine (1805), codeine (1832) and thebaine (1835).^5–7^ This was followed by the development of more potent and efficacious semi-synthetic opioid medications (synthesised in the laboratory from naturally occurring opium compounds), which included diamorphine (heroin) (1874), oxymorphone (1914), oxycodone (1916) and hydrocodone (1920).^8–13^ Although first developed as an antitussive, the euphoric and well-being effects associated with heroin soon became sought after for non-medical purposes, leading to concerns from the early 20th century about dependence.^14,15^

### Legislation to address controlled use

Following the introduction of the Hague Opium Convention in 1912, which obliged signatories to limit the manufacture, sale and use of heroin and morphine to primarily medical and scientific uses, international legislation moved towards stricter controls. The Geneva Convention of 1925 placed further controls on the production and international trade of heroin^16–18^ and alongside the introduction of the Limitation Convention in 1931, a significant decrease was observed in manufacture and consumption.^19,20^ The Single Convention on Narcotic Drugs (1961; and subsequent conventions in 1971 and 1988) placed opioids (and precursors) including heroin, methadone, morphine, and opium into Schedule 1, representing ‘substances with addictive properties, presenting a serious risk of abuse’ and subject to the strict international controls. Despite these international controls, clandestine organisations responded to the profitability of the then controlled opioids, leading to the development of an illicit international market in drugs.^21–23^

### Creation of synthetic opioids

Pharmaceutical companies continued with the development of synthetic opioids, defined as ‘synthesised in the laboratory without the use of naturally occurring opium compounds’, throughout the 20th century for human and veterinary medicine, leading to the discovery of meperidine^24^ in 1939 (with a different chemical structure to morphine but with similar pharmacological properties), followed in 1946 by the synthesis of methadone.^25^ In 1959, fentanyl was developed and became a leading analgesic and anaesthetic agent due to its higher potency relative to morphine (50–100 times greater) and heroin (25 times greater), quicker absorption, and shorter time for onset of effects.^26–29^ However, these properties also made it attractive for non-medical use, and fentanyl and its analogues soon appeared on the controlled market and were thereafter controlled under the UN Single Convention in 1961 (under Schedule I, and for those with no medical utility, Schedule IV).^30^ More recently, over the last decade, new synthetic opioids (e.g. carfentanil and ocfentanil), including non-pharmaceutical products, have been implicated in an international opioid crisis and the associated increase in unintentional overdoses, poisonings, and drug-related deaths.^31–35^ The growing number of synthetic opioids on the controlled drug market, the ability for potent products to be easily transported in relatively small amounts (such as *via* the postal service) and their associated morbidity and mortality, means that they pose a serious and complex challenge to global public health.^36,37^

### Mortality rates

In North America, illegally manufactured fentanyl and other synthetic opioids have significantly contributed to a rapidly worsening disease burden of the ‘opioid overdose crisis’.^38–40^ A ‘triple wave’ of opioid deaths in the United States has been reported, with an increase in mortality related to prescription opioids in the late 1990s, a rapid increase in heroin-related deaths beginning in 2010, followed by fentanyl and other synthetic opioids from 2014 onwards.^32,41^ These are not discrete waves, but overlie each other and all contribute to the overall deaths within each wave. The Centers for Disease Control and Prevention (CDC) reported that 100,306 total drug overdose deaths occurred in the 12 months to April 2021 in the United States, and that synthetic opioids were the main cause of these deaths (75,673; 75.4%). In Canada, 7224 opioid-related deaths were reported in the 12 months to March 2021, an increase of 95%, with the large majority involving fentanyl or other synthetic opioids.^42–44^ The UK Advisory Council on the Misuse of Drugs (ACMD) also published their report on synthetic opioids during the same time,^45^ highlighting that the rates of drug-related deaths had steadily increased over the past decade, and that those related to novel synthetic opioids were likely to be under represented, due to the lack of available detailed forensic analyses. However, in the United Kingdom, the proportion of deaths related to fentanyl and new synthetic opioids is reported to be much lower than in North America. For example, 2020 death registrations estimate there were 60 fentanyl, fentanyl analogue or new synthetic opioid-related deaths, compared with 1337 heroin and morphine-related deaths.

### International response

In response to an emerging global public health crisis, the United Nations Office on Drugs and Crime (UNODC) launched an integrated strategy in 2018 to support countries in addressing the ongoing global synthetic opioids issue, which included coordinating the international response, reducing supply through changes in the scope of control of substances, and promoting effective prevention strategies and treatment options for substance use disorders.^46^ In addition to traditional prevention and treatment responses, strategies proposed by other policymakers, organisations, and researchers in markets with high risk of exposure to new opioids include the expansion of supervised drug consumption facilities; increased coverage of an expanded set of opioid agonist therapies; and the introduction of community-based ‘drug checking’ that would provide rapid information on circulating substances in a local market.^42^

### Appearance of new synthetic opioids

In 2020, the UNODC reported that the number of new synthetic illicitly manufactured opioids identified annually had increased significantly from just one in 2009 to 55 in 2018.^47^ In addition, they reported that between 2015 and 2019, the number of synthetic opioids, as a proportion of NPS, quadrupled from 2% to 8%.^48^ The European Monitoring Centre for Drugs and Drug Addiction (EMCDDA) reported similar findings, and since 2009 a total of 57 new synthetic opioids have been detected for the first time in Europe, including eight reported for the first time in 2019. In contrast to previous years, only two of these were fentanyl derivatives, the remaining six all being chemically distinct from fentanyl, despite posing similar concerns in respect to their toxicity, and they were termed non-fentanyl opioids.^49,50^ In the United Kingdom, the ACMD concluded that synthetic opioids posed a significant risk to public health and recommended that current monitoring and surveillance systems be adapted to help identify the true scale of the public health threat.^51^

### Emerging markets

Synthetic opioids are sold not only as standalone products but also as counterfeit opioid medications and may be adulterants in street-level supplies of controlled drugs such as heroin, cocaine and benzodiazepines.^52^ Although some people will intentionally seek out these synthetic opioids, many are not aware of the constituent elements of what they have purchased and so can be unintentionally exposed to substances of unknown pharmacology and toxicity.^53^ Although financial profit motivates production and distribution, research suggests reasons for emergence may differ among regional markets. In the United States, for example, analysis suggests that growth in synthetic opioid consumption arose after restrictions were placed on access to prescription opioids after the first wave of opioid deaths in the early 2000s, whereas in some European countries this was a result of heroin shortages.^42,50,52^

## Mechanism of action

The endogenous opioid system consists of three opioid receptors: mu-, delta- and kappa-opioid receptors, all of which are 7-transmembrane domain, G-protein coupled inhibitory receptors. Mu-opioid receptors are expressed throughout the peripheral and central nervous system and are associated with analgesia and dependence formation, in addition to their euphoric, sedative and respiratory depressant effects and constipation.^54,55^ Agonism [the combining of a chemical substance (such as a drug) with a specific receptor on a cell thereby initiating the same reaction or activity typically produced by the binding of an endogenous substance] at both delta- and kappa-opioid receptors is also associated with analgesia,^55,56^ and additionally, agonism at kappa-opioid receptors is responsible for the dysphoric effects of opioids, which may also contribute partly to dependence formation.^55,57^ Expression of opioid receptors in humans is most concentrated within the limbic system, hypothalamus, caudate nuclei, periaqueductal grey, dorsal horn of the spinal cord and dorsal root ganglia, and they are found on both pre- and post-synaptic membranes.^56,58^ At the spinal level, opioid receptors work to inhibit afferent nociceptive signalling from the dorsal horn.^56^ Observations of opioid receptors expressed on peripheral sensory neurons, and the effectiveness of peripherally administered opioid analgesia, support the notion that peripheral opioid receptors play an important role in pain perception following injury.^59,60^

The four canonical endogenous opioid receptor ligands are beta-endorphin, leu-enkephalin, met-enkephalin and dynorphin. These ligands are agonists at opioid receptors, each with varying affinities to the three opioid receptor subtypes: beta-endorphin notably acting as a full-agonist of all three opioid receptors, whereas the enkephalins show a relatively higher affinity for delta-opioid receptors, and dynorphin shows a higher affinity for kappa-opioid receptors.^58^

Stimulation of the mu-opioid receptor promotes the exchange of guanosine triphosphate (GTP) for guanosine diphosphate (GDP) in the G-protein complex, which then inhibits adenylate cyclase in the cells causing a decrease in intracellular cyclic adenosine monophosphate (cAMP).^61^ The activation of the mu-opioid receptors also inhibits calcium and potassium ion channel conductance.^62^ These events lead to neuronal membrane hyperpolarisation and inhibition of tonic neural activity, and subsequent reduction of the release of several neurotransmitters (including acetylcholine, noradrenaline, substance P, GABA, and dopamine).^63^

Synthetic opioids stimulate limbic and midbrain dopaminergic circuitry, thought to underpin the euphoric effect sought by users, in addition to also causing depressant effects such as analgesia, sedation, and a reduction in consciousness level.^64,65^ Direct stimulation of the chemoreceptor trigger zone in the area postrema may cause nausea and vomiting.^66^ The toxicity associated with synthetic opioids relates partly to their high affinity for the mu-opioid receptors and their lipophilicity,^67–69^ which can result in the development of opioid toxicity at very low doses.

## Mode of use and clinical presentation

Synthetic opioids are manufactured in powder, tablet (including lozenges), transdermal patch and liquid forms and can be consumed by swallowing, nasal insufflation (snorting), smoking, injecting, transdermal application, or application sublingually, vaginally or rectally. Reported novel ways of consuming these compounds include inhaling using electronic nicotine delivery (vaping) devices. The absorption of synthetic opioids from swallowed transdermal patches can be increased by chewing prior to swallowing. In addition, they can be extracted from transdermal patches for use by alternative routes such injection or nasal insufflation, as the patches retain a large amount of drug even after they have been used therapeutically.^70,71^ Similar to other natural and semi-synthetic opioid use, the main desired effects are relaxation, euphoria and analgesia, but synthetic opioids produce significant inter-individual dose/response variability leading to different toxic doses and clinical presentations.^72,73^ Synthetic opioid affects all the major biological systems, producing effects including nausea, vomiting, bradycardia, hypotension, constipation, weight loss, chest pain, hypoxia, pulmonary oedema and cyanosis.^74–76^ The most common adverse neurological effect is a reduced level of consciousness.^77^ People who have consumed novel non-fentanyl compounds present with a wide range of non-opioid expected adverse effects including paraesthesia, limb weakness, balance disturbance, visual and hearing impairments and skin rashes.^78,79^

## Compound-specific chemical structure

Synthetic opioids include fentanyl (discovered in 1959)^27^ and its analogues used in medical therapy, sufentanil (1974),^80^ alfentanil (1976)^81^ and remifentanil (1987).^82^ Those fentanyls not approved for human medical use are sometimes described as non-pharmaceutical fentanyls and include acetylfentanyl (1962),^83^ carfentanil (1974),^84^ ocfentanil (1984)^85^ and furanylfentanyl (1986).^86^ New synthetic opioids chemically unrelated to fentanyl (non-fentanyl compounds) have emerged on the global drugs market since 2010 and include MT-45 [1-cyclohexyl-4-(1,2-diphenylethyl) piperazine], AH-7921 [3,4-dichloro-N-{[1(dimethylamino) cyclohexyl]methyl} benzamide] and U-47700 [3,4-dichloro-N-[(1R,2R)-2-(dimethy-lamino) cyclohexyl]-N-methylbenzamide].^72,87–89^ Table 1 outlines synthetic opioids, their receptor affinity and potency related to morphine. We now describe some of the more commonly available synthetic opioids, in terms of their pharmacological profiles and where appropriate, atypical unwanted effects reported to be associated with their use.

**Table 1.** Synthetic opioids.

Class	Drug name	Opioid receptor selectivity			Relative potency compared with morphine	Blood concentration found to be lethal (ng/ml)
		μ	κ	δ		
Fentanyl and pharmaceutical analogues	Fentanyl	+ + +			50–100	0.3–383
	Sufentanil	+ + +	+	+	1000–4000	27
	Alfentanil	+ + +			72	100–200
Illicit fentanyl analogues	Acetylfentanyl	+ + +			15.7	153–260
	Acrylfentanyl	+ + +			170	1.86 ± 3.08
	3-methyl-fentanyl	+ + +			48.5–569	0.3–1.9
	β-hydroxy-3-methyl-fentanyl	+ + +			6300	NA
	α-methyl-fentanyl	+ + +			56.9	3.1
	α-methyl-acetyl-fentanyl	+ + +			3.1	NA
	4-fluoro-fentanyl	+ + +			15.7	0.24 ± 0.21
	Butyr-fentanyl	+ + +			1.5–7	0.1–99
	Carfentanil	+ + +			10,000	0.1–4.9
	Isobutyrylfentanyl	+ + +			NA	NA
	Ocfentanil	+ + +			90	5.3–15.3
	Furanyl-fentanyl	+ + +			7	0.4–26
Non-fentanyl analogues	U-47000	+ + +			7.5	13.8–490
	AH-7921	+ + +	+		1–1.7	31–6600
	MT-45	+ + +	+	+	~1	8.3–1989

“+/++/+++” are markers of approximate increasing affinity for the relevant receptor subtype.

### Fentanyl and its analogues

Fentanyl and its main analogues alfentanil, sufentanil, and remifentanil are used in surgery as adjuncts to anaesthesia, for sedation and the treatment of acute and chronic pain.^90^ Fentanyl is a 2-phenylethyl-substituted 4-anilinopiperidine derivative carrying a propionylamide moiety linked to the aniline-nitrogen. There are four structural features which may be modified, resulting in a huge variety of fentanyl analogues: (a) the piperidine ring, (b) the anilinophenyl ring, (c) the 2-phenylethyl substituent, and (d) a carboxamide moiety linked to the anilino-nitrogen.^91–94^

**Fentanyl** [N-phenyl-N-[1-(2-phenylethyl) piperidin-4-yl] propanamide] is a lipophilic phenylpiperidine synthetic opioid, that selectively binds to the mu-receptor in the central nervous system.^27,92^ Its highly lipophilic nature enables it to pass through the membranes easily (including the blood brain barrier) and be widely distributed within the body.^92^ Bioavailability depends on the route of administration. Low oral bioavailability is observed for fentanyl, with intranasal and oral bioavailability being 89% and 50%, respectively.^93^

**Sufentanil** (N-[4-(methoxymethyl)-1-(2-thiophen-2-ylethyl)piperidin-4-yl]-N-phenylpropanamide] is also a phenylpiperidine synthetic opioid. It differs from fentanyl through the addition of a methoxymethyl group on the piperidine ring (which increases its potency and reduces the duration of action) and the replacement of the phenyl ring by thiophene.^80,93^ It is highly selective for the mu-receptor site, and 5–15 times more potent than fentanyl.^94^

**Alfentanil** N-[1-[2-(4-ethyl-5-oxotetrazol-1-yl)ethyl]-4-(methoxymethyl)piperidin-4-yl]-N-phenylpropanamide is also a phenylpiperidine synthetic opioid. It had also been created by introduction of additional substituents into the fourth position of the piperidine ring of fentanyl, in this case with the introduction of a methoxymethyl group coupled with replacement of the phenyl ring of the phenethyl with a tetrazolyl ring.^30,95^ While not as potent as fentanyl, it is about 30 times more potent than morphine.^95^ Compared with fentanyl and sufentanil, it has the most rapid analgesic onset and time to peak effect as well as the shortest distribution and elimination half-life, a small volume of distribution, greater binding to plasma proteins and less lipid solubility.^96,97^

**Remifentanil** {methyl 1-(3-methoxy-3-oxopropyl)-4-[phenyl(propanoyl) amino]piperidine-4-carboxylate} was created by the replacement of the phenyl ring of the phenethyl group in the first position of piperidine ring with a substitution for a carbomethoxy group.^82^ With an analgesic potency similar to fentanyl, it is metabolised directly in the plasma by non-specific esterases, an active group of enzymes found in blood and tissues throughout the body, resulting in an ultra-short duration of action.^98,99^

### Non-pharmaceutical fentanyls

Carfentanil and ocfentanil are two potent synthetic opioids that have been implicated in the international opioid overdose crisis, particularly in the United States and some European countries.^100–103^ Acetylfentanyl and furanylfentanyl have also recently been associated with cases of overdose and death in the United States.^104–111^

**Carfentanil** [methyl 1-(2-phenylethyl)-4-(N-propanoylanilino)-piperidine-4-carboxylate] is a member of the N-4 substituted fentanyl analogues, carrying an additional methyl-carboxylate moiety at the 4-position of the piperidine ring. It is one of the most potent opioids and is approved for use in veterinary medicine only as a general anaesthetic agent or as a tranquillising agent for large animals such as elephants, as its extreme potency makes it inappropriate for use in humans. It has a quantitative potency approximately 10,000 times that of morphine and 100 times that of fentanyl.^68,108,112,113^ Carfentanil is a very potent agonist at all opioid receptors but acts primarily on the mu-opioid receptor subtype. Carfentanil has been found to be mis-sold as other drugs, including heroin, or used as a substitute to reportedly increase profitability, leading to hundreds of opioid overdoses, many of them fatal. It is the most potent opioid present in the controlled market at present.^114–116^

**Ocfentanil [**N-(2-fluorophenyl)-2-methoxy-N-[1-(2-phenylethyl)-piperidin-4-yl] acetamide possesses a methoxy group instead of a methyl group and a fluorine atom on the ortho position of the aniline group.^117^ Ocfentanil was found to be 2.5 times as potent as fentanyl and around 200 times as potent as morphine.^118^ Ocfentanil was never developed for pharmaceutical use and was detected on the controlled drug market after 2010.^114^

**Acetylfentanyl** [N-Phenyl-N-[1-(2-phenylethyl)-4-piperidinyl] acetamide] is the acetyl amide analogue of fentanyl with a substitution of the N-propionyl moiety for an acetyl moiety.^119^ It demonstrates some similarities with heroin such as colour, consistency and pharmacologic activity and is around 15 times more potent than morphine, but it has 3 times lower potency than fentanyl.^120,121^ Reports have suggested the use of propylene glycol electronic cigarettes filled with acetylfentanyl, as well as its mixture with alcoholic beverages as innovative methods for consumption.^122,123^

**Furanylfentanyl** [N-(1-(2-phenylethyl)-4-piperidinyl)-N-phenyl-furan-2-carboxamide] has a furanyl ring in place of the methyl group adjacent to the carbonyl bridge and has a comparable potency to fentanyl.^124^ In a study into fentanyl and analogue-related deaths across five counties in New York between 2013 and 2017, 417 deaths were found to have been reported, increasing from 10 cases in 2013 to 184 cases in 2017 and that furanylfentanyl was one of the common drugs involved.^125^

### Non-fentanyl opioids

Since 2010, a new generation of synthetic opioids, structurally different from fentanyl, have emerged on the recreational drug market. Their chemical structures belong to benzamide (U-47700, U-48800 or AH-7921), acetamide (U-50488, U-51754) or piperazine (MT-45) classes of compounds.^108^

**U-47700** [3,4-dichloro-N-[(1R,2R)-2-(dimethylamino)yclohexyl]-N-methylbenzamide] is a structural isomer of AH-7921. Slang terms include ‘fake morphine’ or ‘U4’and is sometimes referred to as ‘pink’, because of impurities during its production cause the constituent powder to be pink in colour.^126^ It is 7.5 times more potent than morphine, with an affinity for the mu-opioid receptor,^127,128^ and has been associated with recent intoxication cases and deaths in the United States.^129–132^

**U-50488** [trans-3,4-dichloro-N-methyl-N-[ 2-(1-pyrrolidinyl) cyclohexyl]-benzeneacetamide] is a kappa-opioid receptor agonist, with some reported mu-opioid receptor respiratory antagonist effects.^124,133^ Studies in animals have shown that U-50488 causes diuresis and dysphoria rather than respiratory depression or constipation.^134^ The toxicological profile of U-50488 currently remains under research, but the structural similarity to U-47700 suggests that it might pose a significant risk.^135^

**MT-45** [1-cyclohexyl-4-(1,2-diphenylethyl)piperazine] is structurally distinct from other therapeutic opioids and demonstrates selective mu-opioid receptor agonism, with considerably lower delta- and kappa-opioid receptor affinities, and with similar potency to morphine.^136,137^ Reported adverse effects include hair depigmentation and loss, hearing loss, folliculitis, dermatitis, disorganised keratinisation, and bilateral secondary cataracts requiring surgery.^138,139^ MT-45 has been associated with reports of fatal intoxications in Europe.^140^ In Sweden, it was been associated with 28 analytically confirmed deaths between November 2013 and July 2014.^141^

## Management of acute toxicity and overdose

### Clinical features

Fentanyl and its analogues have a high affinity for mu-opioid receptors, which account for the central nervous system and respiratory depression associated with their significant morbidity and mortality.^142^ Typical symptoms seen in an overdose are miosis (‘pinpoint pupils’), respiratory depression, and a decreased level of consciousness or coma. This is known as the ‘opioid overdose triad’.^143^ In severe opioid toxicity, this can lead to respiratory arrest and death. Vomiting in the setting of reduced unconsciousness and/or protective airway reflexes can increase the risk of aspiration. Therefore, loss of protective airway reflexes and/or significant central nervous system depression which doesn’t respond to antidote therapy can necessitate intubation for airway protection. Prolonged admission to intensive care for ongoing management has been reported, in part due to the pharmacokinetic properties of some of these synthetic opioids and their longer duration of action.^144,145^ Other reported unwanted effects seen with synthetic opioids include alterations in muscle tone, chest wall rigidity, ‘seizure-like’ activity, confusion, affective changes, cough suppression, orthostatic hypotension, urinary urgency or retention, folliculitis and dermatitis with hair loss, dry eyes, elevated liver enzymes and delayed bilateral hearing loss.^70,71,78^ Nasal burn or nasal drip after insufflation and a bitter taste after oral ingestion have been reported; these effects are commonly seen with a range of NPS including non-opioid NPS.^64^

### Clinical management

During an overdose, there is a sustained effect on the brainstem and cortical centres regulating respiratory rate, resulting in respiratory depression and potentially death.^146^ Initial management should focus on protecting the airway and maintaining breathing and circulation as in any emergency situation.^147–149^ Naloxone is a competitive mu-opioid receptor antagonist, which reverses central and peripheral opioid effects rapidly. Naloxone can be administered *via* the intravenous, intramuscular, intranasal, intraosseous, subcutaneous, endotracheal, inhalational and sublingual routes.^150,151^ It is recommended that where possible it is given intravenously, as this allows titration of dose to the desired clinical response while reducing the risk of unwanted effects such as acute withdrawal. In the pre-hospital setting or where intravenous access is not possible, then use by intramuscular injection would be appropriate, although there is greater potential for unwanted effects and acute withdrawal due to unpredictable absorption of the naloxone. There has been increasing interest in the use of intra-nasal naloxone in the pre-hospital setting to reduce the risk of needle-stick injuries related to intramuscular injection. The discussion of appropriate dosing regimens in different settings and/or patterns of acute toxicity is outside the scope of this review article, and we recommend that readers obtain this information from their local poisons centre or information services. However, it is worth noting that the high potency, rapid onset of action and relatively long half-life of synthetic opioids pose particular challenges for reversal by naloxone. Reports suggest that the management of a synthetic opioid overdose requires larger or more frequent repeated doses of naloxone than would normally be recommended.^152–157^

## Dependence and withdrawal syndromes

Opioid dependence involves a cluster of symptoms, including impaired control over use, prominence of use of a substance in a person’s life, and physiological symptoms such as tolerance and withdrawal.^39^ It is best characterised as a typically chronic, relapsing condition with periods of active use, abstinence, and relapse over years or decades.^158,159^ Risk of mortality from overdose is increased when tolerance is reduced after a period of abstinence, such as imprisonment.^160–162^ Available data suggest that repeated use of fentanyls and their analogues leads to the development of tolerance and dependence more rapidly than with natural or semi-synthetic opioids^163^ and that non-fentanyl opioids are associated with the highest risk of all the synthetic opiods.^72,164,165^ Typical withdrawal symptoms are similar to that of natural and semi-synthetic opioids and include involve sweating, anxiety, diarrhoea, bone pain, abdominal cramps, and shivers with ‘goose flesh skin’ appearance.^64,166^ Restless legs syndrome and psychotic symptoms have been reported to be associated with synthetic opioid withdrawal.^167,168^

Structured drug treatment interventions (e.g. opioid agonist therapies, psychosocial interventions) are effective in treating opioid use disorders, and pharmacotherapies (e.g. methadone, buprenorphine) reduce the risk of all cause and drug poisoning mortality. Hence, creating opportunities for those who may be exposed to new opioids to access drug treatment is an essential component of a comprehensive strategic response to the emergence of these compounds.^169,170^

## Laboratory testing

Analytical methods for the determination of synthetic opioids are of great importance, and there is a need to focus on identifying both the parent drug and metabolites and to correlate the results with clinical outcomes and intoxication symptoms.^171–173^ The constant arrival of new synthetic opioids on the controlled drugs market presents an important challenge. Most of these new substances are not detected by routine screening and confirmation methods, and due to the low doses of the highly potent drugs, the concentrations expected in the biological samples are in the low ng to pg/ml or ng to pg/g range, requiring extremely sensitive methods of analysis.^174,175^ Routine screening is not undertaken in clinical practice as the results do not change outcome and are not available in a timeframe to change the outcome.

Current robust methods to identify synthetic opioids, due to their enhanced sensitivity and specificity, include gas chromatography coupled with mass spectrometry (GC-MS) or liquid chromatography coupled to tandem mass spectrometry (LC-MS/MS), which allow both qualitative and quantitative analyses in different biological matrices.^176–178^ GC-MS has historically been the analyser of choice in toxicology and a gold-standard test for drug detection and quantification.^179^ However, there are several limitations which include sample preparation that may require several post-extraction derivatisation steps after micro-extraction of the sample has taken place, and a limited capacity for detection of non-volatile or polar molecules, for example, in the context of excretion of hydrophilic metabolites in urine.^131,174,180–182^ LC-MS/MS permits the analysis of polar molecules, with limited sample preparation steps and lower limits of detection, and is therefore prioritised for the analysis of synthetic opioids.^183,184^

Both GC-MS and LC-MS/MS require up-to-date reference libraries for identifying synthetic opioids and face challenges from the continuous emergence of new structural derivatives.^184^ Recent advances in mass spectrometry have permitted the development of high resolution (LC-HRMS) methods, with both targeted and untargeted workflows for synthetic opioid identification.^185^ High resolution mass spectrometry (time-of-flight, Orbitrap) offers potential advantages to identify unknown compounds without the availability of a reference standard, but this technology is not readily available in most forensic laboratories.^185^

## Conclusion

The market in controlled drugs is dynamic and continuously and rapidly changing. NPS producers create new chemical variations offering dangerous new alternatives to drugs that have become restricted, in part so as to circumvent existing national and international drug controls.

The evolving international opioid overdose crisis poses a new threat to the global public health community through the emergence of new synthetic opioids. These substances are readily produced by clandestine laboratories, distributed internationally, acquired easily *via* Internet sites, cheap to purchase, potentially easier to transport than conventional illicit drugs, relatively easy to use and often undetectable by conventional testing techniques. Many of them are much more potent than existing available opioids which has contributed to the rise in severe morbidity and mortality rates observed with their use.

The major public health concern remains that often users are not aware that they are exposing themselves to these more potent opioids, due to their contamination of both opioid and non-opioid controlled drugs. Current laboratory detection methods may not detect all novel synthetic opioids, and detection in healthcare settings is currently sub-optimal. Global public health systems need to coordinate their response and focus on monitoring and intelligence sharing, primary prevention, healthcare workforce preparation, harm reduction, treatment, and public safety, if opioid-related morbidity and mortality are to be successfully addressed.

## Declarations

**Ethics approval and consent to participate:** Not applicable.

**Consent for publication:** Not applicable.

**Availability of data and materials:** Not applicable.
